# Variation in host susceptibility and infectiousness generated by co-infection: the myxoma–*Trichostrongylus retortaeformis* case in wild rabbits

**DOI:** 10.1098/rsif.2007.1075

**Published:** 2007-06-19

**Authors:** Isabella M Cattadori, Réka Albert, Brian Boag

**Affiliations:** 1 Division of Animal Production and Public Health, Faculty of Veterinary MedicineThe University of Glasgow, Glasgow G61 1QH, UK; 2 Center for Infectious Disease Dynamics, The Pennsylvania State UniversityUniversity Park, PA 16802, USA; 3 Department of Physics, The Pennsylvania State UniversityUniversity Park, PA 16802, USA; 4 Birch Brae, Knapp, Perth and Kinross PH14 9SWUK

**Keywords:** nematode and virus–nematode co-infection, *Trichostrongylus retortaeformis*, myxoma poxvirus, age–intensity relationship

## Abstract

One of the conditions that can affect host susceptibility and parasite transmission is the occurrence of concomitant infections. Parasites interact directly or indirectly within an individual host and often these interactions are modulated by the host immune response. We used a free-living rabbit population co-infected with the nematode *Trichostrongylus retortaeformis*, which appears to stimulate an acquired immune response, and the immunosuppressive poxvirus myxoma. Modelling was used to examine how myxoma infection alters the immune-mediated establishment and death/expulsion of *T. retortaeformis*, and consequently affects parasite intensity and duration of the infection. Simulations were based on the general T_H_1–T_H_2 immunological paradigm that proposes the polarization of the host immune response towards one of the two subsets of T helper cells. Our findings suggest that myxoma infections contribute to alter host susceptibility to the nematode, as co-infected rabbits showed higher worm intensity compared with virus negative hosts. Results also suggest that myxoma disrupts the ability of the host to clear *T. retortaeformis* as worm intensities were consistently high and remained high in old rabbits. However, the co-infection model has to include some immune-mediated nematode regulation to be consistent with field data, indicating that the T_H_1–T_H_2 dichotomy is not complete. We conclude that seasonal myxoma outbreaks enhance host susceptibility to the nematode and generate highly infected hosts that remain infectious for a longer time. Finally, the virus–nematode co-infection increases heterogeneities among individuals and potentially has a large effect on parasite transmission.

## 1. Introduction

The probability of parasite establishment, the period of infection and the subsequent rate of transmission can vary considerably between individual hosts within the same population. Some hosts are both more susceptible and more infectious and carry the infection for a longer period than others (Lloyd-Smith *et al*. 2005; Matthews *et al*. 2006). Furthermore, susceptibility and infectiousness often covary in the same host depending on the past history of infection and the presence of co-infections (Tompkins & Hudson 1999; Cattadori *et al*. 2005, submitted). Indeed, most hosts are inhabited by an infra-community of parasites that exert conflicting tensions on the ability to control infections. Co-infections alter the host immune response (Borkow & Bentwich 2006; Walters *et al*. 2006; Graham *et al*. 2007; Resende Co *et al*. 2007), affect pathogenesis (Hirka *et al*. 1991; Mohsen *et al*. 2002; Mwandumba *et al*. 2004; Druilhe *et al*. 2005) and can complicate the efficacy of vaccination (Elias *et al*. 2006). Fundamental to this is the premise that infection by a second parasite species is sufficient to modify host responses—often the immune response—to the first species, leading to changes in susceptibility and transmissibility, and so introducing nonlinearities into the dynamics and influencing the basic reproductive number of the parasite, *R*_0_ (Graham *et al*. 2007). Additionally, seasonal changes in the pattern of exposure and susceptibility to co-infections contribute to generate hosts heterogeneities, as a result of large variations in parasite intensity over time and between hosts (Cattadori *et al*. submitted).

The study of multi-parasite species interaction, how this relates to our understanding of epidemiology and how this influences the efficacy of control is gaining attention as one of the most important issues in the study of human infectious diseases. There is increasing evidence that co-infections contribute to the severity of some of the most serious human infectious diseases including HIV, malaria and tuberculosis (Khan *et al*. 2001; Corbett *et al*. 2002; Abu-Raddad *et al*. 2006; Shivraj *et al*. 2006; Graham *et al*. 2007). A large effort has been spent to reveal the fundamental molecular/cellular processes involved during a single infection; however, there is now the need for a more rigorous examination of the molecular and epidemiological consequences of co-infections. Theoretical studies have investigated competitive direct hierarchy between parasites (Hochberg & Holt 1990; Read & Taylor 2001; Ganusov *et al*. 2002; Bottomley *et al*. 2005) or interaction between parasites with different levels of relatedness (Davies *et al*. 2002; Lively 2005), but have tended to neglect the mediating role of host immunity or the tissue specificity of the majority of multi-species infections in free-living hosts. Here, we take a simple modelling approach and examine how immune-mediated processes in a co-infection can alter host susceptibility and parasite intensity, and can generate heterogeneities among individuals in a population.

Immune-mediated interactions between parasites may occur through cross-immunity (one parasite enhances immunity to a second parasite) or immunosuppression (a parasite suppresses the immune response to another). Careful examination of these processes in controlled laboratory experiments can help to reveal the precise mechanisms (for a review on helminths, see Behnke *et al*. (2001)). On the other hand, the use of field data to predict the epidemiological importance of intra-host processes in natural co-infections is not trivial. One way of exploring the epidemiology of parasite interactions in natural systems is to examine how co-infections shape the relationship between host age and parasite intensity (or prevalence), and from here estimate their contribution to host susceptibility and the period the host remains infectious. The age–intensity curve for a parasite that induces no acquired immunity is equivalent to a simple birth–death process: intensity increases with no constrains or tends to an asymptotic limit when infection rate is balanced by proportional mortality (Hudson & Dobson 1995). In contrast, if the parasite stimulates an acquired immune response, we would expect an initial increase in parasite intensity with age, a rise to a peak followed by a decrease at older ages as a result of immune-mediated clearance (Woolhouse 1992, 1998; Hudson *et al*. 2006). We postulate that the presence of a second co-infecting species, which has either a positive or negative immunomediated interaction with the first, will change the shape of this relationship. If the second species inhibits the immune effectors targeting the survival of the first species, then the period of infection will be longer, the hosts will be unable to clear the infection and intensity of the first parasite will rise to an asymptotic limit. This change in the age–intensity profile could be clearly seen in individuals that became immunosuppressed due to hormonal changes, such as in reproductive females that experience a periparturient rise in helminth intensity during the breeding period (Soulsby 1965; Cattadori *et al*. 2005). If the impact of the second species is simply to suppress the immune-mediated inhibition to the establishment of the first species, basically increasing host susceptibility to the infection, then intensity will increase much faster and reach a peak at a younger age. Moreover, the rate of decline will be faster if there is no change in the immune-mediated response to parasite survival. Similarly, a co-infection that positively affects both establishment and survival will show a faster increment and a higher asymptote of the age–intensity profile compared with single infections (Cattadori *et al*. submitted). Therefore, we propose that co-infections may have an important role in shaping the age–intensity relationship, the average intensity and duration of the infection, and potentially alter parasite transmission.

One inference from the previous section is that the intensity and nature of the immune response will play an important role in modulating host susceptibility, pattern of co-infection and rate of transmission (Graham *et al*. 2007). Conceptually, the nature of the host immune response is determined by the cytokine environment that drives the response of naive T cells into one of two mutually inhibitory T helper cell types (Coffman 2006). Generally speaking, a pro-inflammatory T_H_1 response dominates during microparasite infections (i.e. viruses and intracellular protozoa) by cellular cytotoxic mechanisms that stimulate the production of type 1 and type 2 interferons (IFN), interleukin (IL)-12 and tumour necrosis factor α (Jankovic *et al*. 2001). A T_H_2 response is usually effective against macroparasite infections (like most gastrointestinal helminths) through the production of antibodies and cytokines such as IL-4, IL-5, IL-9 and IL-13 (Neurath *et al*. 2002; Gause *et al*. 2003). The early polarization towards one of the two responses is a critical phase in the subsequent pathway cascade of effectors (Jankovic *et al*. 2001; Coffman 2006). However, both the T_H_1 and T_H_2 pathways may be involved during the course of a micro- or macroparasite infection as parasites may migrate through different tissues, undergo developmental changes during their life cycle or stimulate different effectors during the infection phase (Maizels *et al*. 2004; Pilione & Harvill 2006). Similarly, in the case of a virus–helminth co-infection, while we may expect a T_H_1 polarization of the system towards clearing the virus, which may resolve with a systemic reduction of effectors towards the helminth, at the local level of tissue specificity to the worm the immune response may still be high and fight the infection.

We have examined the interaction between the virus myxoma and the nematode *Trichostrongylus retortaeformis* in a natural population of European rabbits (*Oryctolagus cuniculus*) to explore how myxoma alters host susceptibility to the nematode infection, affects parasite intensity and duration of infection, and ultimately contributes to hosts heterogeneity. Previous studies support the hypothesis that *T. retortaeformis* promotes a strong acquired immune response, which develops as a function of accumulated exposure of the host to the parasite (Michel 1952*a*,*b*; Cattadori *et al*. 2005, submitted; Cornell *et al*. submitted). Myxoma causes the systemic immunosuppressive disease myxomatosis in the European rabbit (Upton *et al*. 1992; Nash *et al*. 1999) and influences helminth aggregation and intensity (Boag *et al*. 2001; Cattadori *et al*. submitted). In the absence of detailed information on the molecular mechanisms that regulate the myxoma–*T. retortaeformis* co-infection, we have used the general T_H_1–T_H_2 immunological hypothesis for a virus–helminth interaction and developed a demographic infection–immunity model that explicitly allows for within-host immune processes. The behavioural characteristics of the single and co-infection models are discussed in relation to the epidemiology of the multi-species infection.

## 2. The system

The system is based on two common parasites of the European rabbits (*O. cuniculus*): the gastrointestinal nematode *T. retortaeformis* and the poxvirus myxoma. Intensity of *T. retortaeformis* and prevalence of myxoma, determined from an assessment of characteristic internal and external lesions (Best & Kerr 2000), were collected from a population of rabbits sampled with a .22 rifle each month between 1977 and 2002 by walking transects across an area of 400 ha in Central Scotland (locality: Littleton, Dundee; Boag *et al*. 2001). *Trichostrongylus retortaeformis* is a nematode with a direct life cycle and a free-living stage: eggs pass onto the pasture in rabbit's faeces and infection occurs through the ingestion of the third-stage larva in the food. Nematode establishment and maturation into adults takes place in the small intestine and the prepatent period is about two weeks (Haupt 1975; Audebert *et al*. 2002). Infection is seasonal and the majority of infective larvae occur in coincidence with rabbit reproduction that, in our population, extends from April to August (Cattadori *et al*. 2005, submitted). Kittens, newborn rabbits of few weeks old, spend their time in burrows sucking milk from their mother and after about a month emerge and start feeding on the pasture (Macdonald 1984). We found that infections by gastrointestinal helminths were first detected between one- and two-month old rabbits (age class 2) suggesting that these individuals experience the contaminated pasture as parasite–naive individuals, and the probability of a worm infection in the burrows can be considered null. Previous studies, based on statistical analyses and modelling of the relationship between host age and *T. retortaeformis* intensity using this dataset, suggest that rabbits acquire immunity to the nematode infection as a function of cumulated exposure to the parasite (Cattadori *et al*. 2005, submitted; Cornell *et al*. submitted). This conclusion is in accordance with previous findings on experimental infections under controlled conditions (Michel 1952*a*,*b*).

The poxvirus myxoma causes the acute and systemic disease myxomatosis in the European rabbit (Kerr & McFadden 2002). Myxoma has the unique characteristic that while stimulating both an innate and acquired immune response, it is also capable of successfully encoding multiple proteins and viral factors that downregulate the host antiviral T_H_1 response (McFadden *et al*. 1995; Kerr & McFadden 2002). One of these properties is the ability to bind rabbit IFN-γ and inhibit the biological activity of extracellular IFN-γ, a key regulatory cytokine in the host immune response against viral infections (Upton *et al*. 1992). Myxoma is mechanically transmitted by the European rabbit flea (*Spilopsyllus cuniculi*); infection initially occurs in the skin and subsequently moves to the lymph nodes (Zuniga 2002), so there is no direct interaction with *T. retortaeformis*. In our rabbit population, epidemics are seasonal and most of the prevalence occurs from July to January. While we do not have details on the dynamics of infection in our population, data suggest that individuals of all ages are highly susceptible to the virus. Moreover, since myxoma outbreaks occur after the peak of the rabbit breeding season, when the majority of kittens have already been exposed to the worm infection, we assumed that the virus infection always follows the nematode infection. Indeed, only four rabbits were found positive to single myxoma infection over 26 years of data. Finally, since attenuated strains were already detected in the UK wild rabbit populations 2 years after the initial introduction of the lethal strain in 1953 (Hudson & Mansi 1955), we assumed that mild strains circulate in our rabbit population and they allow for individual recovery and lifelong acquired immunity (Fenner & Fantini 1999; Kerr & McFadden 2002). Previous analysis showed that strains of intermediate/low virulence produce virus titres (i.e. virus particles in host skin) that fade out slowly (Fenner *et al*. 1956; Dwyer *et al*. 1990). As such, we may assume that the virus circulates in the host well after the acute chronic infective phase.

Owing to the different epidemiological characteristics of myxoma and *T. retortaeformis*, the first immunosuppresses the immune system (Kerr & McFadden 2002) and the second appears to be immunoregulated (Cattadori *et al*. 2005), and the absence of direct competition between the parasites, we examined the immunomediated epidemiological consequences of myxoma on the dynamics of the nematode infection. The relationship between *T. retortaeformis* intensity and host age was investigated in rabbits with and without myxoma infection sampled for 26 years between July and January, when the virus prevalence is high. Rabbits were classified into eight discrete age classes based on body mass. Three main age categories were identified, kittens (classes 1–3), juveniles (classes 4–5) and adults (classes 6–8); each class represents a one month age increment so a rabbit in age class 8 is eight months old (full details in Cattadori *et al*. 2005). Body mass linearly increased with age in kittens and young rabbits while asymptotically tended towards a constant mass in adult hosts (Cattadori *et al*. 2005). This mass–age classification was supported by further analyses of the relationship between mass and body measurements as well as mass and eye lens weight (Cattadori *et al*. 2005, submitted). Moreover, this classification proved to be consistent also for the myxoma positive individuals (Cattadori *et al*. submitted).

For convenience, we concentrated on male rabbits, whose *T. retortaeformis* intensity does not seem to be affected by changes in breeding status, unlike breeding females that show a distinct periparturient rise in the infection (Cattadori *et al*. 2005, submitted). The sampled rabbit population is infected by a community of parasites that may affect the outcome of the *T. retortaeformis*–myxoma interaction (Lello *et al*. 2004). To identify changes in the age–intensity profiles between multi-infected individuals and dual infected hosts (*T. retortaeformis* plus *Graphidium strigosum*, the two most common nematodes in our rabbit population; this is the smallest worm combination that allows statistical power), a preliminary analysis was undertaken. The age–intensity curves for male hosts infected with multiple and dual helminth infections showed similar profiles both for the *T. retortaeformis* and the myxoma–*T.retortaeformis* co-infected rabbits (see complete details in the electronic supplementary material). As such, we selected the multi-infected male dataset for the July–January period, which provides a larger sample size and greater statistical power.

## 3. The model

To examine how the acquired immune response to a nematode infection is altered by a viral co-infection and to explore the consequences to host susceptibility, time of infectiousness and parasite intensity, we constructed a demographic infection–immunity model that explicitly accounts for the within-host immunological process. The basic model assumes that the development of acquired immunity to *T. retortaeformis* is a function of the accumulated exposure to the free-living infective stages (Cornell *et al*. submitted). Adult parasite intensity *P* increases with the rate of ingestion of larvae, namely the force of infection *f*, and decreases as a function of immune-mediated nematode death and expulsion *I*, and by natural parasite mortality with rate *μ*

(3.1) $$\frac{\text{d}P}{\text{d}t}=f_{t}-(I_{t}+\mu )P,$$

where *t* represents the host age. This model is extended to include the functional forms of the force of infection and the immune components.

### 3.1 Force of infection

The infection rate of *T. retortaeformis* was estimated using the feeding rate, as a function of body mass, and the nematode intensity in myxoma negative rabbits of age class 2, i.e. parasite–naive hosts whose worm intensity can be considered as a direct function of the force of infection (Cattadori *et al*. 2005). The daily food intake of a male rabbit that has access to a natural diet is about 5% of its body mass (Jilge 1974; Harkness & Wagner 1995; Irlbeck 2001). Food intake linearly increases with body mass and over 30 days—to match the monthly age classes—the food accumulated *F* is described as a quadratic function of host age *t*, *F*_*t*_=*ct*^2^ with *c*=53 as a constant parameter. While there may be seasonal differences in the composition and amount of the monthly diet (Sibly *et al*. 1990), we restricted our analyses to the food intake by males in the late summer–winter period and avoided spring–summer changes in herbage quality or breeding-related diet. Knowing the adult nematode intensity in naive male kittens of age class 2 (the mean+s.e., 67.8+32.8, was used as worm monthly average) and assuming that the natural mortality rate of infective larvae from L1 stage on the pasture to adult is 90% (Crofton 1948*a*,*b*; Audebert *et al*. 2002), we were able to estimate the monthly infection rate, *f*, of rabbits from age class 2 to 8 as: *f*_*t*_=*Ct*^2^ with the constant *C*=35 and the force of infection in naive kittens of age class 1 *f*_1_=0. We make the assumption that nematode and myxoma–nematode co-infected rabbits are exposed to the same force of infection, that is, their food intake and diet selection is similar.

### 3.2 Immune response

The age–intensity curve provides a timeline integral of changes in the nematode birth–death process but no insight into the underlying mechanism. Previous studies suggest that the convex age–intensity relationship is consistent with the hypothesis that rabbits develop an acquired immune response to *T. retortaeformis* (Michel 1952*a*,*b*; Cattadori *et al*. 2005; Cornell *et al*. submitted). In general, gastrointestinal helminth infections promote a T_H_2 cellular immune response (Finkelman *et al*. 1997; Gause *et al*. 2003). The event leading to a T_H_2 pathway depends on a number of molecules and effectors specific to each host–parasite system. However, common processes have been identified across different helminth–host systems that result in eosinophilia, goblet and mucosal mast-cell hyperplasia and the production of non-complement fixing antibodies (Gause *et al*. 2003; Maizels & Yazdanbakhsh 2003). The relative contribution of these processes on the establishment and death of the helminths in the host is still poorly known. Moreover, there is no indication that acquired immunity would selectively cause the death/expulsion or modulate the establishment of the worm. Therefore, since no immunological details are available for the rabbit *T. retortaeformis*–myxoma system, we followed the simplified assumption of a T_H_1–T_H_2 immune polarization and explored how the T_H_2 immune response towards the establishment as well as the death/expulsion of the nematode is affected by myxoma. We assumed that the intensity of the acquired immune response T_H_2 depends on the accumulated exposure of the host to *T. retortaeformis* infective stages (both larvae and adults) from age 2 to the current age *t*, namely the cumulated force of infection *f*, as ${\text{T}}_{\text{H}}2_{t}=\alpha \int_{2}^{t}f_{x}\text{d}x$. The efficacy parameter α denotes the ratio between the rate of increase in T_H_2-mediated acquired immune response and the rabbit infection rate. Immunity clears *T. retortaeformis* infection by host age *t*, through two main processes, the intensity of which is expected to be proportional to T_H_2: the reduction of nematode establishment RE_*t*_=*γ*T_H_2_*t*_ and the death *E*_*t*_ and/or expulsion *M*_*t*_ of nematodes *IE*_*t*_=*E*_*t*_+*M*_*t*_=(*β*_*E*_+*β*_*M*_)T_H_2_*t*_; here *γ* and *β*_*E*,*M*_ are constants (Maizels & Holland 1998; Grencis 2001). We assumed that the worm establishment rate exponentially decreases with increasing immune response (Cornell *et al*. submitted), for example, through antibody production. We also assumed that the immune-mediated mortality and expulsion is proportional to the nematode abundance, that is, has the same functional form as non-immune-mediated mortality *μP*. Since the time for an ingested infective third-stage larva to become an adult and reproduce is two weeks, we used the time delay *τ*=2 weeks to describe the effective intake of adult worms contributing to the total intensity. The basic model (3.1) modified to include the new components now becomes

(3.2) $$\frac{\text{d}P}{\text{d}t}=f_{t-\tau }\text{exp}(-\gamma {\text{T}}_{\text{H}}2_{t})-[(\beta_{E}+\beta_{M}){\text{T}}_{\text{H}}2_{t}+\mu ]P.$$

No independent estimates exist for the parameter values *α*, *β*_*E*_, *β*_*M*_ and *γ*, therefore we rearranged them as: *α*(*β*_*E*_+*β*_*M*_)=*β* and *γ*/(*β*_*E*_+*β*_*M*_)=*δ*. Here *δ*=RE_*t*_/IE_*t*_ denotes the ratio of the immune activity towards nematode establishment and the immune activity towards nematode death/expulsion. Using the relation between the force of infection and the strength of the T_H_2 immune response, the final infection–immunity model becomes

(3.3) $$\frac{\text{d}P}{\text{d}t}=f_{t-\tau }\text{exp}\left(-\delta \beta \int_{2}^{t}f_{x}\text{d}x\right)-\left(\mu +\beta \int_{2}^{t}f_{x}\text{d}x\right)P.$$

Myxoma infection causes an acute immune response and the polarization of the system towards the systemic T_H_1 pathway but, at the same time, is also capable of evading the antiviral reaction (Kerr & McFadden 2002). We assumed that rabbits of all age classes are equally susceptible to myxoma infection and that the acute infective phase fades out relatively quickly but the virus circulates in the host for a longer period and individuals can fully recover and acquire lifelong immunity (Fenner & Fantini 1999; Kerr & McFadden 2002). Myxoma alters host susceptibility and intensity to the chronic nematode infection. For simplicity we did not explicitly quantify the duration of the viral event but assumed that myxoma decreases the overall T_H_2 immune activity towards *T. retortaeformis* establishment and death/expulsion. Thus, the parameter *α*, which quantifies the relationship between T_H_2 immune response and rabbit infection rate, becomes *α*_*m*_=*θα*, with *θ*≪1 and similarly, the efficacy of the immune-regulated death/expulsion of the nematode decreases to *β*_*m*_=*θβ*.

The relationship between host age and *T. retortaeformis* intensity in male rabbits with the nematode and rabbits with the myxoma–nematode co-infection, during the July–January period was explored. We examined the general case where myxoma infects rabbits at an early age (age class 2), after they have been infected with *T. retortaeformis*, and carry the co-infection to the adult age. This trajectory of co-infection could be interrupted at any age if the host dies due to concomitant infections or initiated at any age if the host already infected with the nematode is then also infected with myxoma. In this case, worm intensity is expected to increase by host age shortly after the co-infection. A number of different scenarios were examined that alternatively considered variation in (i) the virus-induced modulation of the T_H_2 immune response *θ*, (ii) the force of infection *C*, (iii) the ratio between the establishment and the death/expulsion *δ*, and (iv) the total strength of the acquired immune response *β*. The parameter range for *θ* and *δ* was represented as percentage and ratio, respectively. For example, for *θ*=0 the virus completely (100%) subverts the T_H_2 response to mount an effective response towards the nematode, while for *θ*=0.25 the diversion of the immune response towards the virus is incomplete and the T_H_2 response is 25% of the one observed in virus negative hosts. The force of infection *C* has an estimated value of 35 but different values were also tested. The range of the parameter *β* is bounded by the intensity and host age of the observed peak nematode intensity in single nematode infections (e.g. P^*^=296 at the age class t^*^=5) through the relationship $\beta <f_{t*-\tau }/P*\int_{2}^{t*}f_{x}\text{d}x$. Results were qualitatively discussed.

## 4. Results

### 4.1 Observed patterns of infection

*Trichostrongylus retortaeformis* intensity was consistently higher in male rabbits co-infected with myxoma (mean±s.e. 1255.35±128.97, *n*=115) than rabbits without viral infection (481.10±28.71, *n*=749; generalized linear model (GLM) with negative binomial error distribution *p*<0.001, aggregation parameter±s.e. *k*=0.36±0.02; no significant interactions between type of co-infection and age and/or time, figure 1). The age–intensity profile for the nematode infection increased in young males, peaked around age classes 4–5 and decreased in older rabbits (Cattadori *et al*. 2005, submitted; Cornell *et al*. submitted). In contrast, viral–nematode co-infected hosts exhibited a faster increase in nematode intensity in young rabbits and intensities remained high in older males, indicating that these rabbits were not only more susceptible to infection but also were infected for longer periods, and thus represent individuals with a potentially higher transmission rate (figure 1). Specifically, the initial age–intensity slope using the juvenile hosts (age classes 1–4) and the late slope using the old individuals (age classes 5–8) were compared between types of infection to look for differences in the establishment and death/expulsion of worms with and without myxoma infection. The slope coefficients (from GLMs of intensity versus age) were compared with a *t*-test and consistent differences, between types of infection, were observed both for juveniles (*p*=0.001, d.f.=237) and adults (*p*=0.001, d.f.=623), supporting the prediction that myxoma has an effect on the establishment as well as the survival of *T. retortaeformis* (figure 1).

To examine how the seasonal outbreak of myxoma influences the variability in the nematode infection between hosts, the age–prevalence of worm and myxoma–worm co-infected males was examined for the July–January period, during myxoma occurrence, and the February–June period when myxoma is rarely present. *Trichostrongylus retortaeformis* is the most common nematode in this sampled rabbit population and prevalence was always high in rabbits of every age class (figure 2). The prevalence of myxoma–nematode co-infected rabbits represented less than 30% of the sampled population during the viral outbreaks, the middle age classes were more likely to be infected, while fewer kittens and adults were found positive. These results suggest that viral-induced mortality is probably high in naive and adult males; the latter having a higher probability of being multi-infected than the young ones (figure 2). These findings indicate that while the overall prevalence of *T. retortaeformis* infection in the rabbit population is always relatively high, seasonality in myxoma outbreaks and age-related viral mortality contribute to enhance between-host heterogeneity to the nematode infection.

### 4.2 Mechanistic model

Overall, the model that best described the observed pattern of *T. retortaeformis* infection by host age appeared to be represented by the parameter combination *θ*=1, *δ*=0.5, *β*=0.001, *C*=35 and *μ*=0.1 (figure 3). The intensity of adult nematodes increases fast in young male rabbits as a function of the force of infection; however, the slope is less steep than the force of infection partly due to delay in development but also because there is resistance to infection. The acquired immune response initiates with the establishment of the first worms and by around age 5 the model indicates a significant reduction in larval establishment coupled with an increase of immune-mediated death/expulsion of adults, a condition that leads to a decrease in worm intensity and finally to clearance of most of the infection in adult rabbits (figure 3). Nematode natural mortality *μ* (range tested 0, 0.1, 0.2) seems to have a very low impact on the adult worm population compared with acquired immunity (results not shown). Co-infection with the immunosuppressive myxoma alters the ability to control *T. retortaeformis* intensity (figure 3*a*). However, to be consistent with the observed age–intensity profile of the nematode–viral co-infection, the model has to include some immune-mediated regulation of the nematode (0<*θ*<1). In fact, while allowing higher worm intensities in juveniles and adult hosts compared with males infected with the nematode only, the presence of a mild immune T_H_2 component (*θ*=0.25) avoids the exponential uncontrolled increase of the worm intensity as in the case of *θ*=0; thus, we used *θ*=0.25 in the remaining simulations (figure 3*b*–*d*).

Irrespective of the type of infection, an increase in the establishment–death/expulsion ratio *δ* has no clear effect on the age–intensity slope of young rabbits, but decreases *T. retortaeformis* intensities in old hosts and shifts the host age at the peak of infection towards the younger age classes (figure 3*c*). This is probably caused by the nonlinear co-variation between the establishment $f_{t-\tau }\text{exp}\left(-\delta \beta \int_{2}^{t}f_{x}\text{d}x\right)$ and the death/expulsion $-\beta \int_{2}^{t}f_{x}\text{d}x\;P$, as well as the two weeks delay in the prepatent period *τ*. The best agreement with the observed worm intensity data was obtained for *δ*=0.5, that is, the immune contribution to the death/expulsion is twice the immune contribution to the reduction of nematode establishment. Similarly and as expected, an increase in the total immune response of the parameter *β*—which includes both immune-modulated establishment and death/expulsion—decreases worm intensity in older hosts and shifts the age at the peak of infection towards younger rabbits, but has a weak effect on the initial slope of the profile (figure 3*d*). The small change in the initial slope of the age–intensity profiles, observed with different parameter combinations, is probably caused by the relatively weak immunological response of young rabbits to the initial worm infection; this response builds up later with the accumulated exposure to the parasite. However, an increase of the force of infection through the parameter *C* causes a clear shift of the host age at the peak of infection towards the younger age classes and a change of both the initial and late age–intensity slopes. This pattern is consistent with the peak shift hypothesis of a change in the acquired immune response due to changes in host exposure to the infection (figure 3*b*).

## 5. Discussion

We have used an eco-epidemiological approach to the study of concomitant infections and explored how myxoma can modify the host response and the pattern of infection to *T. retortaeformis* in an age-structured wild population of European rabbits. Our objective was to identify the parsimonious mechanism that may explain changes in host susceptibility and time of infection, and ultimately examine how this may generate variation between hosts in the intensity of infections. Our findings support the evidence that the immunosuppressive myxoma virus disrupts the immune regulation of *T. retortaeformis* infection in the absence of any direct competition between the two parasites. However, results indicate that the subversion is not complete and some immune-mediated nematode regulation has to be included in the co-infection model to be consistent with the observed age–intensity relationship. Our findings are also consistent with the hypothesis that myxoma enhances host susceptibility to *T. retortaeformis* and generates highly infected hosts that potentially can remain infectious for a longer time period compared with virus negative hosts. Therefore, if these highly infected individuals also produce more infective *T. retortaeformis* stages over a longer period, co-infections can potentially have a consistent impact on transmission and persistence of the nematode infection in these rabbits (Graham *et al*. 2007). Moreover, since the pattern of the viral–nematode infection changes seasonally, the rabbit population experiences large variations in nematode intensity and prevalence between hosts of different ages, a condition that contributes to increase the persistence of infections (Cattadori *et al*. submitted).

While we have not demonstrated that immune-mediated co-infection is indeed the mechanism driving the system, modelling provides a good insight and support for this hypothesis, and makes quantitative predictions that can be tested experimentally. To reinforce the hypothesis of an immunosuppressive effect of myxoma on *T. retortaeformis*, consider the rise of nematode intensity in female rabbits prior to parturition—the periparturient rise (Cattadori *et al*. 2005, submitted). This phenomenon is caused by a relaxation of immunity in breeding females probably due to hormonal changes (Marquardt *et al*. 2000). We found that the possible reduction in immunity due to the viral co-infection is similar to the reduction of immunity in the periparturient rise of female rabbits.

In the absence of data, we did not explicitly study the effects of time delay between nematode and virus infection, or changes in host susceptibility to the nematode once recovered from myxoma. We have modelled the age–*T. retortaeformis* intensity relationship as a phenomenon that changes as cumulative exposure to the nematode infection. Myxoma acts as a discrete event that alters the dynamics of nematode infection. Myxoma polarizes the system towards a systemic T_H_1 immune response, but a moderate T_H_2 activity is necessary to control the establishment and death/expulsion of the nematode population. Previous studies on wild and laboratory myxoma-infected rabbits showed that both T_H_1 and T_H_2 mechanisms can simultaneously take place in rabbits but this results in significant disease and high mortality rates (Kerr *et al*. 2004).

The sampling of myxoma–nematode infected males from our rabbit population suggests that both parasites can coexist but probably with a conflicting challenge of the immune system and high costs for the host. Another possibility is that the general T_H_1–T_H_2 mechanism of regulation is too simplistic and more complex mechanisms, not necessarily related to the T_H_1–T_H_2 polarization, operate locally at the level of parasite–tissue specificity. The immunosuppressive virulence factors encoded by the virus may in fact allow myxoma to evade the immune response but also downregulate the clearance of the nematode. Indeed, the coexistence of these two parasites may be the result of a trade-off in the virus–nematode coevolution with the host and their immune factors. Theoretical studies have investigated the evolution of myxoma virulence and the coexistence of different strains in host populations and concluded that, among other factors, a combination of age-dependent resistance and seasonal outbreaks allows the circulation of different strains and consequently the long-term persistence of myxomatosis (Dwyer *et al*. 1990; Aparicio *et al*. 2004). Our study suggests that co-infections contribute to age-related changes in host susceptibility and promote between-host heterogeneity both to the virus and the nematode infection, as observed in the seasonal pattern of age–prevalence. We have aggregated the data from the study area over a period of 26 years and it is very likely that there has been some evolution in myxoma virulence over this time period (Fenner & Fantini 1999). Individuals were classified from their disease characteristics and we are aware that a selection towards avirulent strains and asymptomatic disease features may potentially cause some disease misclassification. Additionally, we do not exclude that helminth infections have potentially contributed to modulate the selection for resistance to the virus but also the host trade-off to multiple parasite infections. While these factors may have interfered, we found some clear seasonal and age-related patterns and are confident that our conclusion still holds.

In summary, we have proposed that myxoma alters the immune response of the rabbit and this affects susceptibility and the lifetime production of infectious eggs by *T. retortaeformis*. While other processes may also be involved and contribute to the age–intensity relationship observed, this possible mechanism was based on the general T_H_1–T_H_2 dichotomy of the immune processes. We applied a modelling approach and found that immune-regulated changes in worm establishment and death/expulsion can be used to quantify changes in host susceptibility and time of infection. Co-infections can affect these parameters and consequently the slope and shape of the age–intensity relationship. The next challenge is to precisely measure the development of acquired immunity during a single *T. retortaeformis* infection and the subsequent alterations caused by a myxoma co-infection, and finally examine the hypothesis with accurate immunological parameters and further modelling. We have already started to tackle this issue with field studies and manipulation of the system in the laboratory.

## Figures and Tables

**Figure 1:**
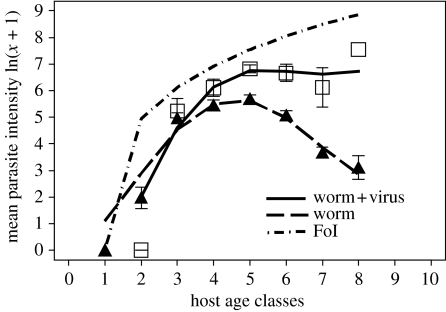
Observed host age–parasite intensity relationships for *T. retortaeformis* in male rabbits with nematode (triangles) and myxoma–nematode co-infection (squares), averaged (±s.e.) over 26 years of data for the July–January period. A cubic spline curve is fitted to the relationship between parasite intensity (ln(*x*+1)) and host age classes. The estimated maximum force of infection (FoI; *f*_*t*_=*Ct*^2^ with *C*=35) by host age is reported by the dotted–point line.

**Figure 2:**
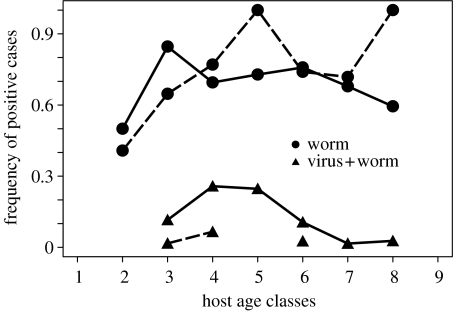
Observed age–prevalence relationships for *T. retortaeformis* and myxoma–*T. retortaeformis* co-infection in male rabbits between July–January (continuous line) and February–June (dashed line), averaged over 26 years.

**Figure 3:**
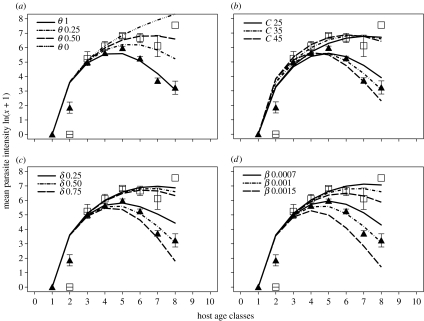
Simulated host age–parasite intensity relationships for *T. retortaeformis* in male rabbits. The basic model is described by the parameters *C*=35, *β*=0.001, *μ*=0.1, *δ*=0.5, *θ*=1 for the nematode and *θ*=0.25 for the worm–virus co-infection. (*a*–*d*) explore the effect of changing one parameter while keeping the others constant, specifically changing (*a*) The strength of the virus-modulated T_H_2 response *θ*, (*b*) the force of infection parameter *C*, (*c*) the immunomodulated establishment–death/expulsion ratio *δ* and (*d*) the strength of the total acquired immunity *β*. The observed mean worm intensity by host age for the nematode (triangles) and virus–nematode co-infection (squares) is reported.

## Supplementary Material

Host age-*T. retortaeformis* intensity in male rabbits with dual and multi-species infections Comparison between datasets
