# Supplementary material for: Variation in host susceptibility and infectiousness generated by co-infection: the myxoma–Trichostrongylus retortaeformis case in wild rabbits
Source: J R Soc Interface. 2007 Jun 19;4(16):831–40. doi: 10.1098/rsif.2007.1075 (PMC2386892; doi:10.1098/rsif.2007.1075)
Supplement: Supplementary file 1 [file rsif20071075s01.doc]

Cattadori et al. Variation in host susceptibility and infectiousness generated by co-infection: the myxoma-*Trichostrongylus retortaeformis* case in wild rabbits

**SUPPLEMENT**

**Host age-*T. retortaeformis* intensity in male rabbits with dual and multi-species infections**

The age-*T. retortaeformis* intensity profile for rabbits with: (i) multi-species infection (5 parasites) and (ii) dual nematode infection (*T. retortaeformis* and *G. strigosum,* the two most common nematode in our rabbit population*;* this is the smallest worm combination that allows statistical power) was examined, to reveal differences in the slope and shape of the profiles between *T. retortaeformis* infection and myxoma-*T. retortaeformis* co-infection. Male rabbits sampled between July and January were selected and we used the approach described in Cattadori *et al.* (2005; under review).

Differences in the age-intensity curves for the nematode and myxoma-nematode co-infected rabbits were consistent when using either multi-species or dual nematode data. In general, intensity increased much faster and reached higher values in myxoma co-infected rabbits compared to myxoma free males, and old age hosts were not able to clear the nematode infection when co-infected with myxoma (Fig. 1s). A comparison of the profiles of these age-intensity curves confirmed the similarities between dual nematode and multi-species data sets. Nematode intensity was consistently higher in viral co-infected rabbits than myxoma negative hosts, both for the dual and multi-species data (for all: P<0.001, see also: Cattadori *et al*. under review). The viral infection altered the age-nematode intensity slope for the juveniles (age class 1 to 4) and adults (age class 5 to 8) both for the dual and multi-species data set. A pairwise t-test of the slope for the same age category (young or adult) and type of infection (nematode or myxoma-nematode) between dual and multi-species data (for example: slope of adults with *T. retortaeformis* in dual data set *vs* slope of adults with *T. retortaeformis* in multi-species data set, etc.) showed consistent similarities for each pairwise comparison (for all: P>0.05, t-test based on slope coefficients from GLMs on nematode intensity *vs* age, for nematode and myxoma-nematode infection). As such, we selected the multi-infected male population, from the July-January period, which provides larger sample size and stronger statistical power.

**Figure 1s.** Age-*T. retortaeformis* intensity relationships in male rabbits with nematode (triangles) and myxoma-nematode co-infection (squares), averaged (±s.e.) over 26 years of data for the July-January period. Two data sets were used: panel **A**- multi-species infected male rabbits (fig. 1 in the manuscript), panel **B**- *T. retortaeformis-G. strigosum* dual infected males. A cubic spline curve is fitted to the relationship between parasite intensity (ln(x+1)) and host age classes.
